# Insights on postneoadjuvant treatment among patients with triple-negative breast cancer and residual disease after neoadjuvant therapy: can expert consensus help to interpret the current evidence?

**DOI:** 10.1038/s41416-026-03452-8

**Published:** 2026-04-29

**Authors:** Antonis Valachis, Jürgen Geisler, Peeter Karihtala, Malgorzata K. Tuxen

**Affiliations:** 1 Department of Oncology, Faculty of Medicine and Health, Örebro University, Örebro, Sweden; 2 Department of Oncology, Akershus University Hospital, Norway & Institute of Clinical Medicine, Faculty of Medicine, University of Oslo, Oslo, Norway; 3 Department of Oncology, Helsinki University Hospital Comprehensive Cancer Center and University of Helsinki, Helsinki, Finland; 4 Department of Oncology, Herlev and Gentofte University Hospital, Herlev, Denmark

**Keywords:** Breast cancer, Chemotherapy, Cancer immunotherapy, Targeted therapies

## Abstract

As treatment strategies for early triple-negative breast cancer continue to evolve, translating clinical evidence into practice can be challenging, especially as the standard of care also shifts and may differ across studies. The management of patients with triple-negative breast cancer who have residual disease after neoadjuvant therapy exemplifies a complex clinical scenario with emerging yet sometimes difficult-to-interpret evidence. Through a critical appraisal of current data and an expert consensus discussion from the Nordic region, the authors present a perspective on postneoadjuvant treatment options for triple-negative breast cancer and discuss potential approaches based on available evidence and expert opinion.

## Introduction

The treatment landscape for patients with triple-negative breast cancer (TNBC) has evolved significantly in recent years, driven by the broader use of neoadjuvant therapy and the adaptation of postneoadjuvant treatment strategies based on tumour response [1]. In addition, new treatment options have emerged, including the use of pembrolizumab in both the neoadjuvant and postneoadjuvant settings, as well as capecitabine and, for patients with germline *BRCA* (g*BRCA*) mutations, PARP-inhibitors, like olaparib, in the postneoadjuvant setting [2–4].

As treatment strategies continue to evolve rapidly, the associated treatment algorithms are becoming increasingly complex due to results from randomised trials that did not incorporate all currently available treatment options. Thus, interpretation often requires clinical extrapolation during decision making, introducing a level of uncertainty that must be acknowledged when communicated to the patients. In this context, guidelines and consensus documents incorporating expert opinions can offer valuable and clinically relevant guidance.

In this perspective article, we present an expert consensus discussion on treatment options in the postneoadjuvant setting for patients with TNBC. We also take into consideration the outcome after neoadjuvant therapy and discuss potential treatment options based on current evidence and available expert opinions.

## Current randomised evidence on postneoadjuvant setting

A summary of the key characteristics and results of randomised trials investigating treatment options in the post-neoadjuvant setting for patients with TNBC is presented in Table 1. The first randomised evidence supporting a successful response-adapted postneoadjuvant treatment in breast cancer originated from the CREATE-X trial [3]. In this study, patients with breast cancer who did not achieve a pathologic complete response (pCR) after standard neoadjuvant chemotherapy were randomised to receive either postneoadjuvant capecitabine for 6–8 cycles or observation. Briefly, the trial demonstrated a statistically significant improvement in overall survival for patients treated with postneoadjuvant capecitabine in the overall cohort (hazard ratio [HR] for death: 0.59; 95% confidence interval [CI]: 0.39–0.90), with a benefit that was limited in patients with triple-negative breast cancer (TNBC) (HR: 0.52; 95% CI: 0.30–0.90).

**Table 1:** Key characteristics of published randomised controlled trials investigating post-neoadjuvant therapeutic interventions in triple-negative breast cancer.

Study name	Main inclusion criteria	*N* of patients	Neoadjuvant treatment strategy	Intervention	Comparator	HR for iDFS	HR for OS
CREATE-X	Stage I-IIIb, non-pCR (no data on gBRCA status)	286^a^	>95% anthracyclines and taxanes (combined or sequential)	Capecitabine x 6-8	observation	0.58 (0.39–0.87)	0.52 (0.30–0.90)
ECOG-ACRIN EA1131	Stage II–III, residual tumour ≥1 cm (no data on gBRCA status)	410	100% taxanes; 85% anthracyclines	Platinum (cisplatin or carboplatin) x 4	Capecitabine x 6	1.06 (0.62–1.81)	1.13 (0.71–1.79)
KEYNOTE-522	Stage II-III (no data on gBRCA status)	462^b^	Carboplatin + paclitaxel x 4 = > AC or EC x 4	Neoadjuvant and postneoadjuvant pembrolizumab for 1 year in total	Placebo		0.76 (0.56–1.05)^b^
OlympiA	Stage II–III, 100% gBRCA-mutation (non-pCR if neoadjuvant therapy given)	1509^a^	>90% anthracyclines and taxanes; 26% platinum-based	Olaparib for 1 year	Placebo	0.62 (0.49–0.79)^a^	0.64 (0.46–0.88)^a^
BRE12-158	Stage II–III, residual tumour ≥2 cm	197	Anthracycline-based and/or taxane-based	Genomically directed therapy	Physician’s choice	0.69 (0.40–1.19)	0.72 (0.38–1.38)

^a^Patients with triple negative breast cancer;

^b^patients with non-pCR.

To further optimise postneoadjuvant treatment, the ECOG-ACRIN EA1131 trial investigated whether platinum-based chemotherapy would be more effective than capecitabine in this setting [5]. Unfortunately, the results did not support this hypothesis. The study reported lower-than-expected invasive disease-free survival across both treatment arms, underscoring the need for improved therapeutic strategies in this high-risk population.

A different approach was explored in the randomised phase II BRE12-158 trial, which evaluated genomically guided postneoadjuvant treatment [6]. Patients with residual disease after neoadjuvant chemotherapy were randomised to receive either physician’s choice therapy (predominantly capecitabine) or targeted therapy based on next-generation sequencing results. However, the genomically directed approach did not demonstrate superiority over capecitabine.

The pivotal KEYNOTE-522 trial established the role of the immune checkpoint inhibitor pembrolizumab in both neoadjuvant and postneoadjuvant settings [2]. The addition of pembrolizumab to platinum, taxane- and anthracycline-based neoadjuvant chemotherapy, followed by continuation for a total of one year, significantly improved overall survival (HR: 0.66; 95% CI: 0.50–0.87). Notably, postneoadjuvant pembrolizumab was administered to all patients, irrespective of residual disease. While the relative risk reduction with postneoadjuvant pembrolizumab was consistent across residual cancer burden (RCB) categories, the absolute benefit was minimal in patients with RCB 0 and greater in those with RCB 1 and 2 [7]. In fact, the absolute difference in 36-month EFS between patients receiving pembrolizumab and those receiving placebo after achieving RCB 0 was 1.9%, whereas the difference in overall survival was 0.7%, corresponding to a number needed to treat of 143 to have one additional patient with RCB 0 alive due to pembrolizumab. Importantly, prognosis remained poor for patients with RCB 2 and especially RCB 3, highlighting the need for treatment escalation in this subgroup. It is worth noting that this study was not designed to explicitly investigate the impact of postneoadjuvant pembrolizumab, as the observed efficacy may largely be driven by the effect of neoadjuvant pembrolizumab on micrometastatic disease prior to surgery. The lack of a survival benefit observed in clinical trials evaluating adjuvant-only treatment with immune checkpoint inhibitors in breast cancer supports this interpretation [8, 9]. In addition, an important consideration when interpreting the results of the KEYNOTE-522 trial is that post-neoadjuvant capecitabine was not permitted.

In the pivotal OlympiA trial, the focus was on patients with germline *BRCA* mutations (gBRCA) and high-risk breast cancer, including TNBC patients with residual disease after neoadjuvant therapy [4]. Patients were randomised to receive 1 year of the PARP (poly ADP ribose polymerase) inhibitor olaparib or placebo. Olaparib significantly improved overall survival (HR: 0.68; 98.5% CI: 0.47–0.97), regardless of tumour subtype, chemotherapy regimen, *BRCA* mutation type (*BRCA1* or *BRCA2*), or the use of carboplatin. In patients with TNBC, olaparib was associated with a 4-year absolute overall survival improvement of 3.8% (90.1% vs. 86.3%; HR, 0.64; 95% CI, 0.46–0.88). Notably, neither pembrolizumab nor capecitabine were allowed as part of the treatment strategy in this trial.

In summary, three randomised trials, CREATE-X, KEYNOTE-522, and OlympiA, have shown positive results for postneoadjuvant treatment escalation strategies in selected patients with TNBC and residual disease after neoadjuvant chemotherapy in terms of prolonged overall survival. However, each trial excluded the other treatment options, leaving room for interpretation and the need to extrapolate findings when determining the most suitable therapeutic strategy for individual patients. Moreover, all three trials underscore the need for improved treatment strategies in this setting, as patients with a poor response to neoadjuvant therapy continue to have an unfavourable prognosis.

## Extrapolating evidence for combination therapies

The key question arising from the randomised evidence and the ongoing need for improved treatment strategies is whether combining therapies in the postneoadjuvant setting could enhance outcomes and maximise clinical benefit.

Since there is a lack of evidence supporting combination therapy in the postneoadjuvant setting for patients with TNBC, any discussion of such strategies must rely on data from the metastatic setting, with careful consideration of potential toxicity risks.

The first combination of potential interest would be to combine pembrolizumab with capecitabine for patients with residual disease after pembrolizumab-based neoadjuvant therapy. Regarding this combination, preclinical evidence suggests that combining capecitabine with pembrolizumab may be beneficial, as capecitabine not only exerts cytotoxic effects but also induces immunogenic cell death, enhances antigen presentation, depletes suppressive myeloid cells, and its metabolite 5-fluorouracil may increase PD-L1 expression, creating a vulnerability that pembrolizumab can exploit [10–12]. Emerging clinical data from the metastatic setting provide additional insights. Several studies have suggested that combining immune checkpoint inhibitors with capecitabine is feasible and does not lead to increased toxicity. Notably, the IMpassion132 trial evaluated atezolizumab in combination with chemotherapy (30% of patients received capecitabine) in patients who experienced disease recurrence within 12 months of prior treatment. The study showed no evidence of increased adverse event frequency with the combination therapy [13]. Similarly, phase II studies in the metastatic setting have reported objective response rates ranging from 13% to 43% when capecitabine was combined with immune checkpoint inhibitors, without a corresponding increase in toxicity [14, 15]. These findings suggest that combining capecitabine and immunotherapy could be a tolerable and potentially effective approach. More insights regarding the safety and efficacy of this combination can be obtained from a phase II study, which aims to be completed by late 2028 [16].

In patients with germline *BRCA* mutations and residual disease following pembrolizumab-based therapy, the combination of pembrolizumab and olaparib in the postneoadjuvant setting appears to be another appealing therapeutic strategy. Although this combination has not yet been evaluated in the early-stage setting, preclinical data and preliminary findings from the metastatic setting support its feasibility and potential efficacy. In preclinical models, PARP inhibition may enhance the efficacy of pembrolizumab by promoting neoantigen release, inducing dendritic cell and T-cell recruitment, and upregulating PD-L1 expression [17–19]. In the MEDIOLA phase II study, patients with metastatic breast cancer and germline *BRCA* mutations treated with olaparib plus durvalumab (another PD-L1 inhibitor) achieved an objective response rate of 63%, with no evidence of increased toxicity [20]. Similarly, in a phase II trial of niraparib combined with pembrolizumab, an objective response rate of 21% was observed across patients regardless of *BRCA* mutation status, again without signs of overlapping toxicity [21]. The randomised phase II KEYLYNK-009 study further reinforced the tolerability of this combination, demonstrating a lower frequency of grade ≥3 adverse events (33% vs. 68%) and fewer treatment discontinuations (9% vs. 20%) for patients receiving pembrolizumab plus olaparib compared to pembrolizumab plus chemotherapy [22].

A relevant clinical dilemma emerging from the CREATE-X and OlympiA trial results is the selection of postneoadjuvant therapy for patients with germline *BRCA* mutations and residual disease following neoadjuvant treatment, as both capecitabine and olaparib represent potentially valid therapeutic options. Several clinical observations support the rationale that olaparib may be the preferred treatment option over capecitabine in this patient population. First, the efficacy of capecitabine in patients with germline *BRCA* mutations in this setting has not been established, as no subgroup analysis based on germline *BRCA* status has been reported from the CREATE-X trial. Interestingly, a post hoc analysis of the FinXX trial (testing the benefit of adding capecitabine to an adjuvant chemotherapy regimen) did not show a definitive benefit of adding capecitabine for patients with *BRCA1*-like tumours [23] whereas a benefit was observed in the patient cohort of unselected TNBC [24]. Moreover, the OlympiAD trial demonstrated that olaparib significantly improves progression-free survival compared to physician’s choice chemotherapy (45% treated with capecitabine) in patients with germline *BRCA* mutation and metastatic disease, with a more favourable safety profile (HR 0.58; 95% CI, 0.43–0.80) [25]. Furthermore, a post hoc analysis of OlympiAD trial comparing olaparib to capecitabine showed a marked benefit for olaparib (HR 0.32; 95% CI, 0.16–0.64) [26].

It is worth noting that the combination of capecitabine and olaparib is not a viable option due to their overlapping toxicity profiles, where the risk of adverse effects outweighs any potential benefit.

## Expert consensus in the Nordics

Considering the complexity of the treatment landscape in the post-neoadjuvant setting among patients with TNBC and residual disease, we conducted a Nordic Delphi expert consensus study focusing on metastatic TNBC to explore the perceptions of clinical experts on how evidence from the post-neoadjuvant setting can be implemented in clinical practice [27]. The Nordic expert consensus panel consisted of 24 breast oncologists from five Nordic countries (Denmark, Finland, Iceland, Norway, and Sweden) with extensive experience in treating patients with breast cancer. The Delphi consensus was conducted as a three-round process, comprising questionnaires in the first two rounds followed by a hybrid meeting in the third round. The consensus agreement threshold was set at 70% and the case vignettes were developed by the Delphi steering committee formed by four oncologists with expertise in breast cancer. During the hybrid consensus meeting, two case vignettes with different clinical scenarios were presented to explore the experts’ perceptions.

The first case vignette described a patient with TNBC and no germline *BRCA* mutation, who received neoadjuvant chemotherapy plus pembrolizumab as part of the KEYNOTE-522 regimen and had residual disease classified as RCB 1. The options given to the experts regarding post-neoadjuvant treatment were pembrolizumab alone, capecitabine alone, a combination of pembrolizumab and capecitabine, or no systemic therapy. The same question and options were posed for the same clinical situation, but the patient had RCB 2-3.

To further explore the potential impact of the presence of a germline *BRCA* mutation on post-neoadjuvant treatment, a second case vignette was presented, in which the patient received the same neoadjuvant treatment as the previous one, but was a germline *BRCA* carrier. The potential treatment options were pembrolizumab alone, capecitabine alone, olaparib alone, pembrolizumab plus capecitabine, pembrolizumab plus olaparib, or no systemic therapy in two clinical scenarios, namely RCB 1 or RCB 2-3.

In the first case vignette, nearly 50% and 74% of the experts would recommend a combination of pembrolizumab and capecitabine for RCB 1 and 2–3, respectively, whereas the rest of the experts would recommend pembrolizumab alone. In the second case vignette, the votes were equally divided among the three options: pembrolizumab plus olaparib, olaparib alone, and pembrolizumab alone for patients with RCB 1. In the alternative clinical scenario with RCB 2-3, nearly 50% of the experts voted for pembrolizumab plus olaparib, followed by olaparib alone, pembrolizumab alone, and pembrolizumab plus capecitabine.

**Fig. 1:**
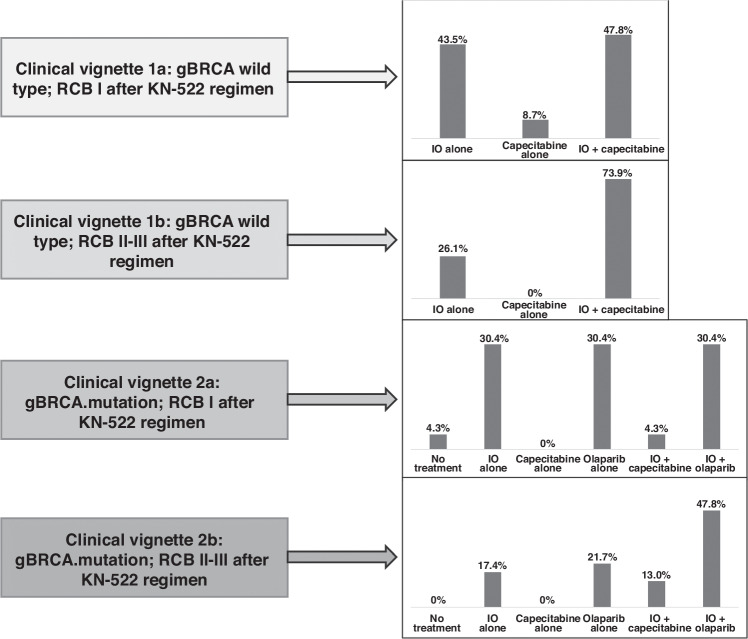
Results from the clinical vignettes at the Nordic Delphi expert consensus project. Abbreviations: RCB residual cancer burden, KN-522 KEYNOTE-522 trial, IO immunotherapy.

Taken together, the results show that no prespecified consensus level of 70% was reached for 3 of the 4 clinical situations, while the fourth reached a consensus slightly above 70%, albeit with strong opposition during the consensus discussion (Fig. 1). To enhance the generalisability of the Delphi results beyond the Nordic countries, experts were instructed to base their votes on the best available scientific evidence rather than on local clinical practice patterns or country-specific reimbursement policies.

## Expert consensus with panel voting regarding postneoadjuvant treatment worldwide: are there any differences?

Several expert consensus recommendations based on panel voting have been published in recent years, and it is of interest to qualitatively compare the level of agreement and potential differences in the interpretation of the available evidence, or its absence.

At the 2023 St. Gallen International Consensus Conference, 62% of the panel voted in favour of combining pembrolizumab and olaparib for patients with germline *BRCA* mutations who had received pembrolizumab-based neoadjuvant therapy and presented with residual disease after surgery. As the benefit of adding post-neoadjuvant capecitabine to patients continuing adjuvant pembrolizumab is unknown, the option of combining pembrolizumab and capecitabine was not addressed by the panel [28].

In contrast, the Breast-Gynecological and Immuno-Oncology International Cancer Conference (BGICC) consensus reported that 73% of panelists supported the combination of capecitabine and pembrolizumab in patients with residual disease and wild-type *BRCA* status, whereas 53% favoured the combination of olaparib and pembrolizumab in patients with germline *BRCA* mutations [29]. Similarly, the expert panel of the Brazilian Society of Mastology reached a consensus of 74.8% for the combination of capecitabine and pembrolizumab in *BRCA* wild-type patients, whereas the level of consensus was considerably lower (51.4%) for olaparib and pembrolizumab in *BRCA*-mutated patients [30]. Offering a different perspective, 84.6% of the panelists in the Asia-Pacific expert consensus on *BRCA*-associated breast cancer agreed that the decision to administer olaparib concurrently or sequentially with pembrolizumab could be considered on a case-by-case basis [31].

## Exploring the reasons for the differences in consensus level

Several factors may influence the level of consensus in clinical situations where robust evidence is lacking. One possible explanation for divergent interpretations is a more conservative approach to evidence. Medical conservatism emphasises the need for strong evidence demonstrating both efficacy and safety before adopting a new treatment strategy [32]. Ensuring safety for a new treatment strategy is particularly relevant in the adjuvant setting, where patients are often already cured of their cancer and the primary goal of therapy is to reduce the risk of recurrence. In such cases, evaluating the potential harms of treatment becomes essential, especially in the absence of randomised data supporting efficacy [33].

However, one could argue that a strictly conservative approach may limit the ability to adapt to an evolving treatment landscape. With multiple clinical trials exploring various therapeutic strategies running in parallel, the definition of standard-of-care can shift during the course of a trial, complicating the interpretation of findings once they are published. Moreover, it is often not feasible to generate randomised evidence for every conceivable clinical scenario. In this context, real-world evidence may serve as a valuable complement, supporting clinical decision-making in complex situations where direct trial data are lacking and treatment recommendations must rely on extrapolation [34].

An interesting observation is the trend toward greater acceptance of combining treatment strategies when both agents are already well-established. Notably, the level of consensus for combining pembrolizumab with capecitabine was generally higher than for the combination of pembrolizumab with olaparib. Capecitabine has been used in the post-neoadjuvant setting since the publication of the CREATE-X trial over 8 years ago, which may contribute to clinicians’ increased familiarity and confidence in its safety profile. In contrast, olaparib represents a newer therapeutic option in this context and is not yet widely adopted in routine practice. In this context, reimbursement strategies and treatment availability across countries may also lead to varying levels of consensus. It can be challenging to recommend treatment strategies that include components unavailable in certain countries, where there is no clinical experience with their use.

## Translating the current evidence and clinical experience into a treatment algorithm

**Fig. 2:**
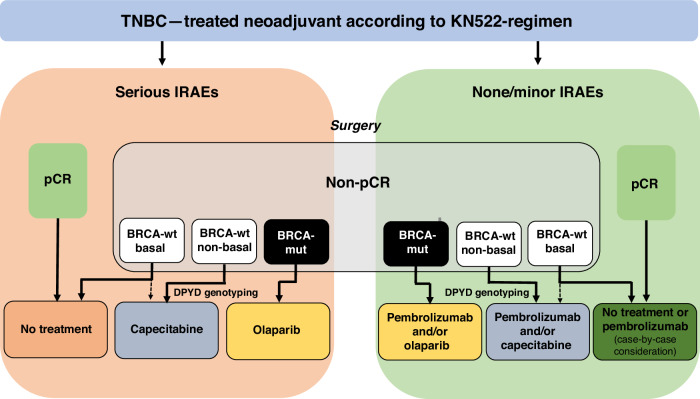
Suggested treatment algorithm for post-neoadjuvant therapy in patients with triple-negative breast cancer who received a KEYNOTE-522-based regimen as neoadjuvant therapy. NOTE. The dashed arrows suggest that there might still be a valid option to discuss postneoadjuvant capecitabine in patients with BRCA-wt, non-pCR TNBC with basal features, as further evidence on the predictive role of TNBC subtyping is pending. Abbreviations: TNBC triple negative breast cancer, KN522 KEYNOTE-522 trial, IRAES immune-related adverse events, pCR pathologic complete response, BRCA-wt BRCA-wild type, BRCA-mut BRCA-mutation.

Figure 2 presents a treatment algorithm for post-neoadjuvant therapy in patients with TNBC who received a KEYNOTE-522-based regimen, based on current evidence, expert consensus, and the authors’ clinical experience. Considering the uncertain absolute benefit of post-neoadjuvant pembrolizumab in patients with pCR, along with the risk of immune-related adverse events (IRAEs) that may impact long-term quality of life [35], the algorithm includes the presence of IRAEs as a key factor in treatment decision-making. The preference for olaparib over capecitabine in patients with gBRCA mutations and non-pCR is supported by data from metastatic breast cancer, whereas the recommendation to consider combining pembrolizumab with olaparib in this setting is based on preclinical and early clinical data, as well as the worse prognosis observed in KEYNOTE-522, particularly in patients with RCB 2 or 3. Although this extrapolation of results from preclinical data and from the metastatic to the adjuvant setting is reasonable, it carries an inherent risk of overinterpretation that should be acknowledged.

Regarding postneoadjuvant capecitabine for non-gBRCA-mutated patients with non-pCR, evidence from translational analyses of randomised controlled trials suggests that the benefit of adding capecitabine as adjuvant therapy is driven primarily by the non-basal TNBC subtype [5, 36]. Although such data are not available from the CREATE-X trial, extrapolating these findings to current clinical practice could be relevant as part of efforts to optimise post-neoadjuvant therapy. It is also important to ensure dihydropyrimidine dehydrogenase (DPYD) genotyping before initiating post-neoadjuvant capecitabine, and in cases of complete DPYD deficiency, the treatment strategy should be modified.

## Conclusion

The treatment landscape for patients with TNBC who receive neoadjuvant therapy and have residual disease is rapidly evolving, yet evidence guiding the optimal post-neoadjuvant therapeutic approach remains limited. In this context, expert consensus recommendations can provide valuable guidance for clinicians by highlighting the most relevant treatment strategies. However, interpreting consensus results can be challenging, as they may reflect the subjective views of panelists and vary depending on clinical experience, regional practice patterns, and tolerance for uncertainty. As novel therapies such as antibody-drug conjugates enter the post-neoadjuvant setting, treatment decisions will become increasingly complex, and interpreting emerging data will require careful consideration. As several ongoing studies are investigating different antibody–drug conjugates compared with standard chemotherapy in this setting [37, 38], their results may impact future treatment algorithms and care pathways. Despite these challenges, and while we are waiting for solid data from clinical trials, expert consensus will remain as a useful tool for navigating the present uncertainty in clinical decision-making. Additionally, incorporating real-world evidence may serve as a crucial complement to randomised data, helping to bridge gaps where randomised evidence is lacking and supporting treatment decisions in routine clinical practice.

## Data Availability

The data from the Delphi consensus are available upon request.
